# A Treatise on the Cause of the Disease Called by the People Milk Sickness; as It Occurs in the Western and Southern States

**Published:** 1841-11

**Authors:** 


**Art. I.**—*A Treatise on the Cause of the Disease called by the People Milk Sickness; as it occurs in the Western and Southern States.* By John Simpson Seaton, M. D., of Jefferson County, Ky. Louisville: pp. 49.

In this little work Dr. Seaton has sought to establish the theory, that Trembles and Milk Sickness are caused by arsenic, or, rather, some of its preparations, dissolved in spring-water. His arguments may be reduced to two. 1st. The prevalence of the disease in certain parts of the state of Indiana, where nodules of iron pyrites containiug arsenic, are found; 2nd., the resemblance of the symptoms of Milk Sickness to those produced by poisoning with arsenic. Dr. S. has not shown, that the water of the springs where his observations were made, contains arsenic or any other poisonous impregnation; nor even that arsenical pyrites is found in all the localities in which the Milk Sickness occurs. Nor has he traced out an analogy between the symptoms of Milk Sickness, and those from poisoning by other substances, as the preparations of barytes, zinc, copper and lead.

We admit that Milk Sickness *may* arise from arsenic, but such possibility, might be met, by alleging the possibility, that it *might* be produced by many other causes. A theory cannot be established without positive facts, but such facts our author has not yet adduced.

1st. The symptoms which characterize Trembles and Milk

Sickness do not bear a closer resemblance to those occasioned by arsenious acid, than they bear to the symptoms of poisoning by various corrosive, acrid, and narcotico-acrid substances—mineral and vegetable. Guided by the same analogy, those maladies have been ascribed to the salts of lead, and to mushrooms. The truth is, that when the stomach is the seat of a severe irritation, the symptoms which result from it, must necessarily be nearly the same, whatever may be the remote cause.

2nd. Dr. Seaton has found nodules of arsenical pyrites in certain slate and sandstone formations where the Trembles prevail. But in the state of Ohio, the *very same formations are entirely exempt from that disease.* This is the case, from Mount Vernon in the interior of the state, down to Portsmouth, at the juncture of the Scioto river with the Ohio. East of this line to the Muskingum, and beyond, there are coal, slate and sandstone strata, to the exclusion of almost every other rock, but Trembles and Milk Sickness are unknown.

3rd. Our author seems to admit, that the limestone formations are free from arsenical nodules, and affirms that they are equally exempt from those diseases. The former may be true—*the latter is at direct variance with fact.* Those maladies prevail in the valleys of the Kentucky and Licking rivers, in this state; in the valley of White Water, Indiana; and in those of the Great and Little Miamies, and all the western tributaries of the Scioto, in the state of Ohio, though no rock but limestone is found in all those districts.

4th. In this Journal, for March, 1841, we have shown on the highest possible authority, that in certain parts of Ohio, the cultivation of the soil prevents the further occurrence of those diseases, although the water of the springs and ponds of course remains unchanged, as it respects a mineral poison.

5th. We have, also, shown in the same paper, that while the geological constitution of adjoining portions of the district in which our observations were made, is the same, those tracts only, which are level and covered with heavy oak timber, are liable to Trembles and Milk Sickness.

6th. In the same paper, we recorded as an indubitable fact, that of a herd of cattle, a part of which were pastured on a meadow, and part in an adjoining woodland, *while the whole were watered from a common well*, the former remained free from Trembles, but many of the latter died of that disease.

7th. In all parts of the West, where Trembles has prevailed, the disease has constantly diminished with the clearing and cultivation of the country; an effect which could not occur, if it depended on mineral impregnation of the water.

These facts in the absence of *all* positive evidence in favor of the arsenical hypothesis, requires us to reject it till further proof is adduced. Such proof Dr. S. expects to develope by a course of experiments, and we hope that he will make them. That he can destroy both health and life, in man and beast, by the administration of arsenious acid, is quite certain. That the stomach will be the chief seat of the morbid action, is equally certain. But would the disease thus induced, be, *of course*, identical with Milk Sickness and Trembles? We think not. It *might* be, and it might *not*. To settle the identity, several things must be established. *First.*—The symptoms must be the same, in the *particular*, not in the *general*. *Second.*—The morbid appearances after death must be the same. *Third.*—The quantity administered, in a given time, must equal what the animal would take by drinking the water of a spring impregnated with the poison. *Fourth.*—The milk of a cow taking the poison, must produce Trembles in her calf and Milk Sickness in those who drink it. *Fifth.*—The flesh of animals dying under experiment with the Trembles, must, when eaten by dogs, raise the same disease in them. *Lastly.*—A preparation of arsenic must be detected in the water of a spring, which was used by animals before being attacked by the Trembles, and which were so confined that other causes could not have acted then. These various points being settled affirmatively, the arsenical theory would be established. The spring whose waters, as ascertained by the proper tests, are impregnated with salts of arsenic, would then be avoided, and the disease disappear. This avoidance is all that would result from the discovery, for springs thus empoisoned, could neither be made pure nor their deleterious properties neutralized.

But would not the same remark apply to all the remote causes which have yet been proposed? Undoubtedly. Suppose it should be discovered, that the disease arises from malaria—how could it be prevented, any more than we can prevent bilious fever? Should it be found to arise from mineral exhalations—how could they be arrested? Should a forest-plant be convicted of the mischief—how could it be eradicated, without destroying its associates by the axe and the plough? We cannot but regard the search after the special cause of Trembles and Milk Sickness as promising very little of real utility. The ascertainment of the infested localities should be our great aim. They might then be avoided, or have their surface transformed by the hand of art. In the state of Ohio, the latter has been found infallible; and we know of no fact going to prove that the same process would not be effectual every where.

We cannot refrain from saying that an undue degree of importance has been attached to the disease or diseases we are now considering. The mortality from them is very small, compared with that from many other maladies about the causes of which we make but few inquiries. There can be no doubt that more persons, annually, die in the West, from autumnal fever, than have died of Milk Sickness, from the commencement of its settlement. Even in the districts where the disease is endemic, it does not destroy as many as pleurisy or cholera morbus.

In conclusion we ought to observe, that there are physicians who deny that the disease called Milk Sickness, is a specific malady; and insist that it is nothing more than autumnal fever of a congestive type, attended with great irritability of the stomach. D.

